# New Method for Obtaining a Bioactive Essence Extracted from Iberian Ham Fat Rich in MUFA and Antioxidants

**DOI:** 10.3390/molecules27020428

**Published:** 2022-01-10

**Authors:** Eva Bruna-García, Beatriz Isabel Redondo, Marta Miguel Castro

**Affiliations:** 1 Department of Bioactivity and Food Analysis, Institute of Food Science Research (CIAL, CSIC-UAM), 28049 Madrid, Spain; evabruna1@gmail.com; 2 Research and Development Department, Cárnicas Joselito S.A., 37156 Guijuelo, Spain; 3 Animal Production Department, Faculty of Veterinary, Complutense University of Madrid, 28040 Madrid, Spain; bisabelr@pdi.ucm.es

**Keywords:** Iberian ham, by-product, extraction, healthy fats, functional ingredient

## Abstract

Iberian ham is one of the most representative Spanish products and presents an excellent nutritional and sensory quality. Iberian ham trimming fat is considered a by-product and to give a new use to this remaining part could represent a healthy and innovative option for obtaining sustainable foods. The purpose of this work was to obtain a new bioactive ingredient from Iberian ham trimming fat with the highest amount of antioxidants and monounsaturated fatty acids (MUFA), using a new non-invasive solvent-free method. To obtain the essence, two different extraction procedures were carried out. After fatty acid characterization, degree of acidity, peroxide index and a basic sensory analysis were performed. Antioxidant in vitro activity and total phenolic compounds (TPC) were also determined. This new ingredient showed a better sensory profile than raw ham fat, a lower degree of acidity, a higher content of MUFAs, and also showed a higher antioxidant capacity and an increase in phenolic compounds compared to the raw material. This bioactive essence could be used as a food, a cosmetic or a nutraceutical ingredient to prevent certain diseases related to oxidative stress and could also contribute to the maintenance of the circular economy.

## 1. Introduction

The Spanish meat industry is the fourth industrial sector in Spain and occupies first place in the entire Spanish food and beverage industry, which reached a record figure in 2020 [1]. Pig production is the foremost Spanish meat activity [1,2] and the subsector dedicated to the production, transformation and elaboration of products derived from the Iberian pig represents a group of special relevance in our country and worldwide, from a cultural and gastronomic point of view. Meat products from Iberian pigs are the most representative traditional Spanish meat products, and they are rapidly spreading to the most prestigious “gourmet” markets [3]. To obtain these products, a diet based on grass and acorns is essential [4] and so is a curing time of more than 36 months, which produces the accumulation of high levels of bioactive compounds in its tissues and provides an excellent quality of meat [3,5,6]. Although production of the products obtained from the Iberian pig is very sustainable and has a high yield, there are some parts that are not intended for direct consumption, such as the fat from the ham trimming. This fat is considered a by-product that comes from slicing ham, which is traditionally discarded or in some cases is used to make broths and other dishes typical of Spanish gastronomy. In this context, Iberian ham fat has been described as having a high content of monounsaturated fatty acids (MUFA) and polyunsaturated fatty acids (PUFA), mainly highlighting the levels of oleic acid and linoleic acid [3,7,8] and the lower concentrations of saturated fatty acids (SFA) than those found in hams from white pigs [9]. Various scientific studies have shown that fats with a high content of MUFAs produce a beneficial effect on blood cholesterol, by causing an increase in HDL cholesterol and a reduction in LDL cholesterol. In this way, it contributes to reducing the risk of various cardiovascular diseases [10]. Moreover, natural antioxidants are accumulated in ham, such as vitamin E, which can be beneficial not only for the animal’s but also for human health [7]. Antioxidant compounds present in ham could act as protective agents against excess reactive oxygen species and could neutralize free radicals, inhibit enzymes such as xanthine oxidase, lipoxygenase and NADPH oxidase, and could prevent the cell death or increase the production of endogenous antioxidants [8,11]. Antioxidants can alleviate and/or protect from several diseases related to oxidative stress such as cancer, diabetes, cardiovascular diseases or neurodegenerative diseases [12]. The hypothesis of this study is that the Iberian ham trimming fat could be used to obtain a new bioactive product, which could provide an interesting sustainable and bioactive food. The use of this fat could suppose the revaluation of a by-product of the industry and the generation of a healthy and innovative option which would be framed within the circular economy and the objectives and goals of sustainable development and requirements of the 2030 agenda. The purpose of this work was to obtain a new bioactive ingredient from Iberian ham trimming fat with the highest amount of antioxidants and MUFAs, using a new non-invasive solvent-free method.

## 2. Materials and Methods

**Figure 1:**
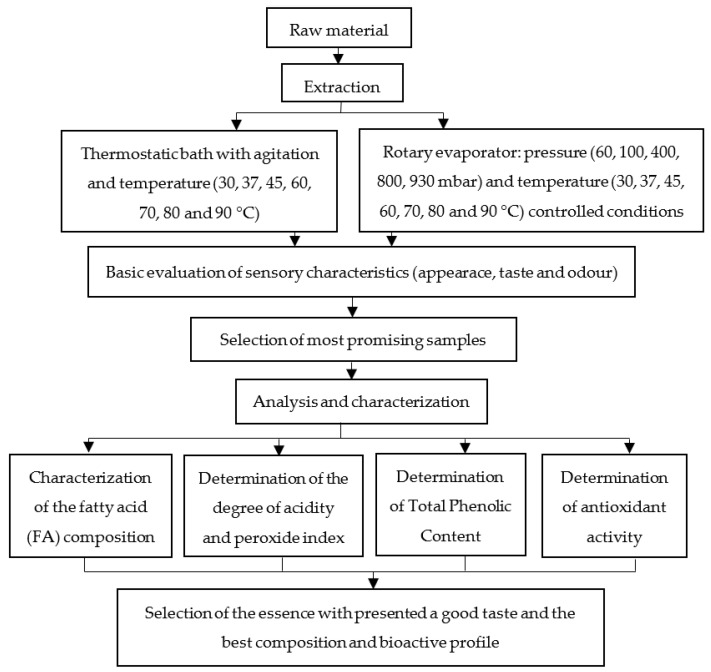
Protocol followed to perform this study.

Figure 1 shows the protocol followed to perform this study.

### 2.1. Raw Material

The ham fat used was provided by the company Cárnicas Joselito S.A. (Guijuelo, Spain), guaranteeing the traceability of each batch. The fat comes from Joselito hams^®^ with a minimum cure of 36 months. The raw material was stored at 4 °C until use.

### 2.2. Extraction Methodology

To obtain the essence of ham fat, two different batches were carried out using two different extraction procedures. On the one hand, a thermostatic bath (Model BTG BUNSEN, Madrid, Spain) with agitation was used. 70 g of fat were added in an Erlenmeyer flask and heated in the bath at different temperatures (30, 37, 45, 60, 70, 80 and 90 °C), for 2 h, 1 h or 45 min depending on the temperature. A higher temperature implied a shorter test time. The extracted essence at each temperature was divided into different aliquots of 2 mL each and were stored at 4 °C and −20 °C for further testing (fatty acid characterization, degree of acidity, peroxide index, antioxidant capacity and total phenolic content). In a second batch, the same temperature conditions were used (30, 37, 45, 60, 70, 80 and 90 °C), but the sample was subjected to vacuum and different pressures using a rotary evaporator (BÜCHI Labortechnik AG, Flawil, Switzerland, 2019) (patent No. P202130236). This equipment was used to carry out an extraction of the essence under vacuum conditions without dissolvent and with controlled pressure and temperature. The established rotating conditions were established at 80–90 rpm, and different pressures (60, 100, 400, 800, 930 mbar) were tested inside the flask at different temperatures to check the performance and the stability of the extraction. In the same way, different aliquots of each extracted essence were stored at 4 °C and −20 °C for its use in same determinations as performed before. For each temperature tested, fat samples from 3 differents pigs were used, and 3 extractions were carried out in each case.

### 2.3. Basic Evaluation of Sensory Characteristics

Basic organoleptic aspects (appearance, color, odor, texture and flavor) were mainly evaluated to make a first discriminatory classification of the products obtained, and those that presented unpleasant tastes and odors were discarded. For this, a basic acceptance test was used, which allows evaluating the acceptability of the product or determining whether one or more samples are more acceptable than others [13]. This evaluation was carried out by 12 man and women participants with an age between 22 and 60 years.

### 2.4. Characterization of the Fatty Acid (FA) Composition

FA analysis was carried out according to the methodology described by Lopez-Bote et al., 2003, based on the extraction and acid methylation of the fatty acid methyl esters for their subsequent injection and determination by gas chromatography. Fatty acids were identified by gas chromatography using a Hewlett Packard 6890 gas chromatograph (Hewlett-Packard Company (HP), Palo Alto, CA, USA) and a 30 m × 0.32 mm × 0.25 µm cross-linked polyethylene glycol capillary column. A temperature program of 170 to 245 °C was used. The injector and detector were kept at 250 °C. The flow rate of the carrier gas (helium) was 2 mL/min [14]. 

### 2.5. Determination of the Degree of Acidity and Peroxide Index

To determine the degree of acidity and the peroxide index of the different samples obtained, the official European analysis methods were used [15]. The two determinations were carried out in triplicate and the results of the degree of acidity are expressed in percentage of oleic acid and the peroxide index expressed in milliequivalents of active oxygen per kg of fat (meq O_2_/kg sample).

### 2.6. Determination of Antioxidant Activity

Antioxidant activity was determined by the Oxygen Radical Absorbance Capacity (ORAC) method, described by Garcés-Rimón et al., 2016 by using fluorescein as a fluorescent substance (ORAC-FL) [16], with some modifications according to Pérez-Jimenez et al., 2008, Zullo and Ciafardini, 2008 and Bataglion et al., 2014, for its application in a lipophilic sample [17,18,19]. All samples were diluted in phosphate buffer (75 mM; pH 7.4) in order to create a concentration curve with the area under the curve (AUC) of each measurement. The reaction was performed in a final volume of 200 μL:20 μL test samples, or 20 μL Trolox solutions (0.2–2 nM) for the calibration curve, 120 μL fluorescein solution (1.17 mM) and 60 μL AAPH (1.3% solution) were added to the wells of a black 96-well plate (Corning, Kennebunk, ME, USA). The fluorescence was recorded at 37 °C every 55 s for 95 min using a fluorimeter (SpectraMax M2; Molecular Devices, San Jose, CA, USA), with excitation and emission wavelengths of 485 and 520 nm, respectively. The extraction of the lipophilic fraction was carried out with 80% methanol. Each of the samples together with the 80% methanol were vigorously vortexed for 2 min and then on a temperature-free plate shaker for 15 min. Then it was centrifuged at 18 °C and 3000 *g* for 10 min, and the supernatant was recovered. Finally, the collected supernatant was diluted in phosphate buffer (PBS 75 mM, pH 7.4) at different concentrations. All samples were measured in triplicate and the results were expressed as μmol Trolox equivalents (TE)/g of essence.

### 2.7. Determination of Total Phenolic Content (TPC)

TPC were determined by the Folin–Ciocalteu method, proposed by Singleton et al., 1999 and updated by Ainsworth and Gillespie, 2007, [20,21] with some modifications for lipophilic samples according to Capannesi et al., 2000 and Seneviratne et al., 2008 [22,23]. The determination of TPC of the samples was carried out in a transparent polystyrene 96 multiwell plate (Corning 9018, Madrid, Spain). 24 µL of sample, standard or water (reagent blank) and 47 µL of Folin’s reagent (diluted 1:10 in water) (Merck, Darmstadt, Germany) were added to each well. Next, 189 µL of sodium bicarbonate (700 mM, Sigma-Aldrich, St. Louis, MO, USA) was added to each well. After incubation at room temperature for 2 h, the absorbance at 765 nm (BioTek Synergy HT, Winooski, VT, USA) was evaluated. Gallic acid (Sigma-Aldrich, St. Louis, MO, USA) was used as a standard in a concentration range of 20–1500 µM. The equation of the standard line obtained was used to calculate the amount of TPC. The extraction of the lipophilic fraction was carried out with 80% methanol. Each of the samples, together with 80% methanol, were vigorously vortexed (Labolan MX-S 51988, Navarra, Spain) for 2 min and then on a stirrer plate (IKA Labotechnik, KS125 Basic, Staufen, Germany) without temperature for 15 min. The samples (Eppendorf AG, Hamburg, Germany) were then centrifuged at 18 °C and 3500 *g* for 12 min, and the supernatant was recovered for the assay. All samples were measured in triplicate and the results were expressed as mg equivalents of gallic acid (GAE)/100 g of the sample.

### 2.8. Statistic Analysis

Results were expressed as mean ± standard deviation (SD). The data were analyzed with the Excel program (Microsoft Office, Washington, WA, USA) and the statistical program Graph-Pad Prism 7.00 for Windows (Graph-Pad Software, San Diego, CA, USA). One-way ANOVA was used as statistical test, where a value of *p* < 0.05 indicates that there are significant differences between two values.

## 3. Results and Discussion

### 3.1. Obtaining Different Essences from a New Method of Ham Fat and Assessment of the Basic Sensory Characteristics

To date, there are few studies that use ham from acorn-fed Iberian pigs as an object of study, and most of the studies have been carried out with cured ham [24,25]. The studies carried out so far in acorn-fed Iberian pigs have focused, for the most part, on evaluating how acorn intake affects the increase in the amount and proportion of monounsaturated fatty acids in the ham [4] and in its relationship between acorn and the aromatic profile of ham [26]. However, until now, by-products from Iberian hams have not been investigated. 

In order to evaluate all the samples that had been extracted with both techniques (thermostatic bath and vacuum), a basic sensory analysis was carried out and the color, taste, smell, texture and general appearance were analyzed. Table 1 shows the results obtained according to the temperature and extraction time in the thermostatic bath and rotary evaporator. The sensory results obtained were similar among all study participants. The samples obtained in a thermostatic bath, presented unpleasant, oxidized and rancid flavors and odors, something that had already been initially detected during the obtaining of the different essences at different temperatures. After this analysis in the thermostatic bath, it was decided to eliminate the samples obtained in the thermostatic bath for the rest of the study, since they did not meet the pre-established sensory expectations and had mainly musty odours. Table 1 shows the results obtained by vacuum and different pressures using a rotary evaporator. With the use of a rotary evaporator, it is possible to obtain the extraction of a sample under vacuum conditions without solvents, since the rotary evaporator includes a vacuum system that reduces the internal pressure of the system. In a preliminary study that was carried out, it was observed that the most suitable pressure to achieve the extraction of the essence was 100 mbar. It is important to have carried out this study to be able to know what is the lowest pressure that can be used to extract the essence, since a lower pressure in the system, that is, vacuum, means that the extraction point is lower, and this means that the sample should be heated for less time, as this may affect its sensory properties. The results showed that the samples extracted at 100 mbar, which presented the best results, were those extracted in a rotary evaporator at 30, 60 and 80 °C. The essence obtained at 30 °C presented finesse in the mouth, but the sample at 60 °C presented a greater taste and smell of ham. On the other hand, the sample extracted at 80 °C showed a certain bitterness, but was pleasant on the palate and with an interesting cellar/dryer aroma. With the results of the samples obtained in the rotary evaporator for all the ham fat samples obtained, it was decided to select the three samples obtained at 30, 60 and 80 °C for subsequent analysis as they had the most pleasant, intense and moderate ham aroma.

### 3.2. Characterization of the Fatty Acid Composition, Determination of the Degree of Acidity and Peroxide Index

FA composition was characterized in the three samples extracted under vacuum conditions at 30, 60 and 80 °C. Table 2 shows the results obtained for saturated (SFA), monounsaturated (MUFA) and polyunsaturated (PUFA) fatty acids from the different samples. The results obtained did not show significant differences in the concentration of total SFA, MUFA and PUFA between the essences extracted at 30 and 60 °C, but differences were observed when the essences were compared with the raw material (Table 2). Lipolysis occurs throughout the ripening process and extraction conditions of the essence [27] and is presumably for PUFA decrease. Moreover, the decrease observed in oleic acid, as a consequence of its degradation, could release aromatic compounds, such as el octanal y nonanal, responsible of the fruity flavor [28].

Significant differences (*p* < 0.05) were found in the stearic and oleic compositions of the three extracted essences compared to the raw material. Moreover, it can be observed that the lower values of SFA (30.02%) and the higher values of MUFA (61.94%) and PUFA (8.04%) correspond to the essence obtained at 60 °C; therefore, this sample seems to present a better composition of FA with respect to essences extracted at 30 and 80 °C. Previous studies with other animal fats indicate that subcutaneous goat fat, beef fat and lamb fat have higher levels of SFA (50.55, 55.15 and 68.05%, respectively) than the analyzed samples (30–32%), and chicken fat possess similar SFA levels (32.48%) [29,30]. Regarding the content of MUFA and PUFA, the essence of Iberian ham fat presents values (67.66–69.98%) similar to those of chicken fat (67.52%), and higher than those of lamb fat, goat and beef (31.95%, 43.90% and 44.85%, respectively) [29,30,31]. The proportional distributions of the individual fatty acids of the analyzed essences showed that the oleic acid content (58.12 ± 0.25, 57.62 ± 0.90, 56.96 ± 1.78%, respectively), represents the greater amount of total MUFA (60.6–61.82%), and these compounds are related to an antihypertensive effect and prevention of cardiovascular diseases [10,32,33]. This MUFA composition is higher than that obtained in other types of animal fats, such as beef (35.70%) [30] or goat (36.16%) [29]. In addition, linoleic acid can also stand out as the predominant PUFA and total PUFA value (7.06–8.04%) presents a higher value than beef fat (6.59 ± 0.61%) and mutton fat (1.61 ± 0.06%) [30]. The dietary intake of PUFA, and especially linoleic acid, is associated with a reduced incidence of cardiovascular disease (mainly coronary artery disease) and metabolic syndrome or type 2 diabetes [32,34].

The degree of acidity and the peroxide index of the three samples of ham fat essence were analyzed (Table 3). Lipids are susceptible to hydrolysis, oxidation, and other chemical processes that result in the production of various compounds. The activity of these lipolytic and oxidative enzymes can lead to the formation of strange tastes and odors [35].

The degree of acidity is a measure of the amount of free fatty acids in the oil [36]. The results obtained from essences extracted at different temperatures (30, 60 and 80 °C) expressed in percentage (%) of oleic acid, do not present significant differences (*p* < 0.05) (29.88 ± 3.8, 24.69 ± 0.91, 26.19 ± 0.75, respectively) when they were compared to the raw material (28.94 ± 1.31%). In extra virgin olive oil, the degree of acidity is considered to be a crucial aspect, which cannot exceed an acidity level higher than 0.8–2% oleic acid [15]. On the other hand, peroxides are products generated by the oxidative degradation of polyunsaturated fatty acids in oil. They build up slowly over time, contributing to the rancidity of the oil, resulting in undesirable tastes and odors. Their identification provides useful information on the preservation of the oil and its rancidity [20].

Table 3 shows the results of the peroxide index. Raw material showed the lowest values in peroxide index and significant differences (*p* < 0.05) were observed between all extracted samples and the raw material. In addition, essence obtained at 80 °C showed the highest value, which suggests that the extraction temperature can influence the peroxide quantity. Alviárez et al., 2016, also observed that the peroxide index was influenced by the extraction temperature used to extract oil from the Amazonian cocoa beans. In this study, 25–27 °C, 50 °C and 70 °C were used, and the peroxide indexes of the extracted oils were 2.98 ± 0.0, 24.91 ± 3.7 and 52.81 ± 0.3 meq O_2_/kg of sample, respectively [37]. In addition to extraction temperature, the lipolysis processes that occur during the curing time of the hams, in our case greater than 36 months, could be related to the degree of acidity and the concentration of peroxides of the essence.

### 3.3. Antioxidant Activity and TPC

The ORAC method is a relevant method because it uses a source of biologically free radicals (peroxyl radical) that is the most prevalent free radical in human biology [38], and is comparable to biologically generated reactive oxygen species (ROS) in the body. The production of ROS, together with the antioxidant defense of the body, can cause oxidative damage. Antioxidant activity is a measure that evaluates the ability of a compound to reduce the impact of ROS [39].

In order to know if the extraction of the essence by means of vacuum conditions at different temperatures influences their antioxidant capacity, the antioxidant capacity of each one of the samples extracted at 30, 60 and 80 °C was evaluated by the ORAC method. Table 4 shows the antioxidant activity of the essences. The highest ORAC value was observed in the essence extracted at 30 and 60 °C, and significant differences (*p* < 0.05) were observed when comparing the ORAC value of these essences with the ORAC value of the raw material. These results suggest that the extraction temperature to which the ham fat is subjected could be a determining factor in its antioxidant capacity. Different studies have shown that if a food is subjected to high temperatures (for example, frying an egg or roasting coffee in the air), there is a decrease in ORAC values [40,41]. In addition, all essences presented a better antioxidant capacity than those obtained in other oils such as peanuts and refined olive oil (1.06 ± 0.09, 1.55 ± 0.08 μmol TE/g, respectively) [42]. More recently, other vegetable oils have shown ORAC values lower than those obtained in our essences. The ORAC values in virgin coconut oil were 0.008 μmol TE/g [43], in soybean oil they were 0.091 ± 0.2 μmol TE/g [44], in palm oil of 0.654 ± 0.5 μmol TE/g [44] and in different avocado oil they were 0.028–0.058 μmol TE g [45]. However, the results obtained are lower than those obtained in different types of extra virgin olive oil samples (3.60 ± 0.15–8 ± 0.1 μmol TE/g) [42,44,46]. It is important to emphasize that the extraction of extra virgin olive oil, in accordance with the regulations, is carried out at a temperature <27 °C, a process known as cold extraction [47,48]. Moreover, in this complex matrix, there are many compounds interacting with antioxidants that are in the cell membrane microsomes, and it has been postulated that the animals fed in “montanera” show a low oxidation and stabilization of the lipids of the microsomal membrane [49]. However, in the case of ham fat there is the presence of sea salt used for curing hams, which is known to be a pro-oxidant agent [50], along with the hemo iron and oxygen [51].

In addition, the TPC values of the essences extracted at 30, 60 and 80 °C were showed in Table 4. The amount of TPC constitutes an important determination since natural phenols improve its resistance to oxidation [52]. The highest TPC result was presented by the essence extracted at 60 °C (139.51 ± 4.84 mg GAE/100 g), obtaining a significantly higher concentration (*p* < 0.05) than raw material. These results suggest that there is a greater TPC release depending on the temperature or the extraction process. Previous studies have identified up to 32 phenolic compounds present in the acorns on which the Iberian pig feeds, such as gallic acid or ellagic acid [49,53]. These compounds could have been stored in fat and could thus be present in the essence studied. These results are significantly higher than other oils such as coconut oil (7.78–29.18 mg GAE/100 g) [54], but lower than those of oil extracted from nectarine seeds (334.5 mg GAE/100 g) [55]. The result obtained in the sample extracted at 80 °C (126.79 ± 3.57 mg GAE/100 g) is similar to the result obtained in the avocado oil (128.17 ± 0.04 mg GAE/100 g) [56], but the sample extracted at 60 °C presents a significantly higher result (139.51 ± 4.84 mg GAE/100 g) than that of avocado oil [56]. Since the phenolic compounds are subject to deterioration when exposed to high temperatures [55], it seems that with an increase in the extraction temperature to 80 °C, there is a decrease in TPC, so that the extraction at 60 °C would be the most suitable for the preservation and release of TPC and also showed better ORAC values. It has been studied that Iberian pig pigs fed mainly with acorns and grass provide MUFA, a high amount of antioxidants and phenolic compounds [57,58]. This feeding strategy affects the chemical composition and oxidative stability of pork tissues, increasing the amount of tocopherols and phenolic compounds [57].

## 4. Conclusions

In the last century, many efforts have been made to preserve the Iberian breed to achieve a sustainable food with high nutritive and sensory qualities. Based on the philosophy of zero waste, low carbon footprint and circular economy, in the present study we have used a by-product from fat obtained after slicing of Iberian ham to produce a bioactive essence through a 100% natural process, using vacuum extraction under a controlled pressure and temperature. The bioactive essence showed a lower degree of acidity than the starting fat, which means that it has a lower proportion of free fatty acids and a lower degree of hydrolysis, which produces a reduction in rancidity of the product. The essence also demonstrated a high content of MUFA, highlighting its oleic content and showing a higher antioxidant activity and total phenolic compounds compared to the raw fat. Regarding all of the results, the essence obtained using vacuum extraction with controlled pressure (100 mbar) and temperature (60 °C) presented a good taste and the best composition and bioactive profile. This bioactive essence could provide beneficial effects from the point of view of health, well-being and improvement of the quality of life as a food ingredient, cosmetic or nutraceutical, and also could contribute to reducing the oxidative stress and other related pathologies. We are aware that a better and deeper biochemical and sensory characterization of the essence will be necessary, as well as new experiments that demonstrate its in vivo effect before being marketed.

## 5. Patents

The results of this study are included in patent No. P202130236.

## Figures and Tables

**Table 1:** Sensory results obtained after subjecting the ham fat to different temperatures in a thermostatic bath and rotary evaporator.

Extraction Temperature (°C)	Extraction Method			
	Thermostatic Bath		Rotary Evaporator	
	Colour of Essence	Odour of Essence	Colour of Essence	Odour of Essence
30	Thick	Dry smell	Yellow	Dry smell, great ham odour
37	Opaque, lumpy in appearance	Pleasant aroma, musty notes	Less thick	Dry smell
45	Less thick	Musty	Viscous	Moderate intensity
60	Bright yellow, transparent	Musty	Yellow	Moderate intensity to ham
70	Bright yellow, presence of lumps	Pungent and musty	Bright yellow	Moderate intensity, musty notes
80	Bright yellow	Pungent and musty	Bright yellow	Pleasant aroma
90	Bright yellow	Putrefied smell	Bright yellow	Pungent and musty

**Table 2:** Fatty acid composition of the selected essences.

	Fatty Acid (%)		Extraction Temperature (°C)		
		Raw Material	30	60	80
SFA (%)	Lauric	0.05 ± 0	0.06 ± 0	0.05 ± 0.01	0.05 ± 0.01
	Myristic	0.94 ± 0.07	1.10 ± 0.03	1.06 ± 0.09	1.05 ± 0.08
	Palmitic	19.67 ± 0.26	20.25 ± 0.63	19.20 ± 0.80	20.54 ± 1.10
	Margaric	0.20 ± 0.02	0.22 ± 0.03	0.24 ± 0.05	0.23 ± 0
	Stearic	7.56 ± 0.49 ^a^	8.6 ± 0.28 ^b^	9.32 ± 0.20 ^b,c^	10.31 ± 1.12 ^c^
	Arachidic	0.15 ± 0.01	0.13 ± 0.01	0.15 ± 0.01	0.16 ± 0.03
	Total SFA (%)	28.56 ± 0.51 ^a^	30.36 ± 0.32 ^b^	30.02 ± 0.91 ^b^	32.34 ± 2.21 ^c^
MUFA (%)	Palmitoleic	1.95 ± 0.09	2.16 ± 0.03	2.14 ± 0.20	2.12 ± 0.14
	Cis-10-heptadecenoic	0.23 ± 0.02	0.28 ± 0.02	0.38 ± 0.07	0.27 ± 0.03
	Oleic	60.48 ± 1.0 ^a^	58.12 ± 0.25 ^b^	57.62 ± 0.90 ^b^	56.96 ± 1.78 ^b^
	Eicosanoid	1.46 ± 0.06	1.26 ± 0.04	1.8 ± 0.07	1.25 ± 0.08
	Total MUFA (%)	64.11 ± 0.95 ^c^	61.82 ± 0.22 ^b^	61.94 ± 0.70 ^b^	60.6 ± 1.77 ^a^
PUFA (%)	Linoleic	6.74 ± 0.41	7.17 ± 0.10	7.39 ± 0.42	6.51 ± 0.43
	α-linolenic	0.58 ± 0.08	0.65 ± 0.07	0.65 ± 0.12	0.55 ± 0.08
	Total PUFA (%)	7.33 ± 0.48	7.82 ± 0.17	8.04 ± 0.53	7.06 ± 0.51

Mean of three measurements ± standard deviation. Different letters (^a^, ^b^ or ^c^) in the same row show significant differences (*p* < 0.05).

**Table 3:** Degree of acidity (% oleic acid) and peroxide value (meq O_2_/kg fat) of the selected essences.

Extraction Temperature (°C)	Degree of Acidity (% Oleic Acid)	Peroxide Value (Meq O_2_/kg Fat)
Raw material	28.94 ± 1.31 ^a^	2.43 ± 0.62 ^a^
30	29.88 ± 3.80 ^a^	10.11 ± 0.60 ^b,c^
60	24.69 ± 0.91 ^a^	8.56 ± 0.82 ^b^
80	26.19 ± 0.75 ^a^	11.10 ± 1.27 ^c^

Mean of three measurements ± standard deviation. Different letters (^a^, ^b^ or ^c^) in each column, show significant differences (*p* < 0.05).

**Table 4:** Antioxidant capacity and TPC of the selected samples.

Extraction Temperature (°C)	Antioxidant Capacity (μmol TE/g)	TPC (mg GAE/100 g)
Raw material	1.86 ± 0.15 ^a^	73.46 ± 2.23 ^a^
30	2.32 ± 0.08 ^b^	94.04 ± 3.98 ^b^
60	2.36 ± 0.06 ^b^	139.51 ± 4.84 ^c^
80	2.11 ± 0.09 ^a,b^	126.79 ± 3.57 ^d^

Mean of three measurements ± standard deviation. Different letters (^a^, ^b^, ^c^ or ^d^) in each column show significant differences (*p* < 0.05).

## Data Availability

Not applicable.

## Sample Availability

Samples of the compounds are available from the authors.
